# The Role of Vitamin D in Obese Children with Non-Alcoholic Fatty Liver Disease and Associated Metabolic Syndrome

**DOI:** 10.3390/nu15092113

**Published:** 2023-04-27

**Authors:** Mioara Desdemona Stepan, Ștefănița Bianca Vintilescu, Ioana Streață, Mihaela Andreea Podeanu, Dan Nicolae Florescu

**Affiliations:** 1 Department of Infant Care-Pediatrics-Neonatology, University of Medicine and Pharmacy of Craiova, 200349 Craiova, Romania; desdemona.stepan@umfcv.ro; 2 Laboratory of Human Genomics, University of Medicine and Pharmacy of Craiova, 200638 Craiova, Romania; ioana.streata@umfcv.ro; 3 Doctoral School, University of Medicine and Pharmacy of Craiova, 200349 Craiova, Romania; 4 Research Center of Gastroenterology and Hepatology, University of Medicine and Pharmacy Craiova, 200349 Craiova, Romania; dan.florescu@umfcv.ro

**Keywords:** obesity, NAFLD, MS, vitamin D

## Abstract

Non-alcoholic fatty liver disease (NAFLD) represents a complex chronic condition, which in the absence of screening–monitoring markers and effective standardized treatment is one of the most important issues in pediatric pathology. In this study, we analyzed the role of vitamin D supplementation in obese children with/without NAFLD and the impact on the components of the associated metabolic syndrome (MS). The study included 22 children with simple obesity (SO) and 50 with NAFLD, aged between 6 and 14 years, who received regimen-based therapy or vitamin D supplementation in case of deficiency. Anthropometric and paraclinical data associated with MS were statistically compared before and after treatment. It was observed that there was a statistical association of NAFLD with MS components, which were present both in SO and in the 6–9 years group. Vitamin D deficiency was associated with the presence of obesity, NAFLD and MS components, and correction of the deficiency induced a tendency to normalize the associated parameters. In the case of a treatment strictly based on the regimen, we found decreases in vitamin D values and additional alteration of some parameters. Supplementation with vitamin D potentiates the effects of the specific regimen, and the effects seem to be dependent on the MS components.

## 1. Introduction

Non-alcoholic fatty liver disease (NAFLD) is the most common chronic liver disease in pediatric pathology, in the absence of infections, medication or autoimmune processes [1,2,3]. The incidence of the disease is 3–17% in the pediatric population but can increase up to 80% in obese children [3,4,5,6,7,8,9], depending on the diagnostic methods [10,11] and the homogeneity of the studied groups [2,3].

The prevalence increases with age; NAFLD is usually diagnosed after 10 years [5,6], mostly due to the silent clinical aspect [11], and although it is reported that it can occur in children under this age [6], the studies about this issue are relatively few.

Although they are numerous, the studies related to NAFLD for both adults and children are extremely heterogeneous, regarding the criteria for establishing the study groups, the diagnostic methods, the definition of entities such as the metabolic syndrome (MS) and the treatment [2,11,12,13,14], aspects that are also reflected in the results.

NAFLD constitutes a spectrum of sequential entities represented by simple steatosis, non-alcoholic steatohepatitis (NASH) that can evolve into cirrhosis and cancer, the damage to hepatocytes, inflammation and fibrosis being the result of complex multistage two-hit or multihit pathogenic mechanisms, with the involvement of still unclear environmental, genetic and epigenetic factors [1,2,4,5,6,8,15,16,17]. Resistance of adipose tissue to insulin, oxidative stress, intestinal dysbiosis and disturbance of the intestine–liver axis constitute the mechanisms that maintain and advance NAFLD [1,4,11,16,18].

Obesity seems to be an essential condition for the development of NAFLD [2,5,13,19], although there are relatively uncertain data about the existence of so-called lean NAFLD with normal body mass index (BMI) [3,11]. There have been described clear methodological procedures for evaluating the distribution of fat mass such as bioelectrical impedance analysis (BIA) and dual-energy X-ray absorptiometry (DXA) [20]. Adipose tissue plays an active hormonal role [2], and through the lipotoxicity of the pro-inflammatory cytokine/adipocytokine profile, it promotes and makes the transition to metabolic and cardiovascular changes that are considered components of MS [10]. In this way, the mechanisms of obesity, NAFLD and MS are interconnected [2,10,21] as a continuous feed-forward loop. The pathogenic aspects are controversial, and the determinism between the three entities is intensely discussed, as it is unclear which of these are initiating conditions [12,22]. In addition, in the case of children, besides the fact that investigations are rare under the age of 10 years, the presence of MS components in the case of simple obesity (SO) [2] raises the issue of the possibility of NAFLD underdiagnosis [23].

The gold standard considered imperfect [4] for the diagnosis and progression of NAFLD is the liver biopsy, but due to the incidence of the disease, the invasiveness and complications of the technique, the presence of harvesting errors and the price, noninvasive approaches are required, on which there is no consensus related to specificity and sensitivity [2,11,17,24]. Serum alanine transaminase (ALT) and liver ultrasonography are the recommendations for NAFLD diagnosis by the North American Society for Pediatric Gastroenterology, Hepatology and Nutrition (NASPGHAN) [5,6,24]. Currently, there is no consensus regarding the NAFLD evaluation and management algorithm, although serum, genetic or imaging parameters, even some of them, were considered effective (gamma-glutamyl transferase, elastography, probiotics/prebiotics) or having unclear effectiveness (cytokeratin 18, cathepsin D, interleukin-6, patatin-like phospholipase domain-containing protein 3- PNPLA3, adipokine, hepatokine) [4,6,19,25,26].

One of the many therapeutic possibilities that have been approached over time for the treatment of NAFLD is represented by the additional administration (supplementation) of vitamin D, with anti-inflammatory [21,27] and antioxidant effects [23], improving glucose tolerance/insulin resistance [28]. However, the results are controversial [28,29], so at the present time, the only approach that has proven its effectiveness in NAFLD in children is lifestyle improvement [4,7,11,18]. Despite this, there are some pharmacological agents approved by the United States Food and Drug Administration (FDA) for weight loss in children over 12 years old [19].

The heterogeneity of the groups, the unclear pathogenesis, the absence of specific screening, diagnostic and monitoring markers, and the absence of an effective and standardized treatment for NAFLD, including in children, represent a major reason for research in this direction and designate the lesion as a major health problem in pediatric pathology. Thus, through the extrahepatic manifestations and future comorbidities, as well as the numerous investigations and treatments, NAFLAD is considered a condition that consumes immense resources, which are difficult to quantify [30,31].

In this study, we analyzed the relation of vitamin D with the pathogenic sequence of simple obesity (SO)–NAFLD and the MS components and the role that regimen-based treatment or association with vitamin supplementation may have in obese children.

## 2. Materials and Methods

### 2.1. Study Design

The prospective controlled experimental study was designed to investigate the efficacy of vitamin D in the treatment of obese children with or without non-alcoholic fatty liver disease (NAFLD) and with the presence or absence of metabolic syndrome. The patients included in the study were evaluated related to their medical history, epidemiological, clinical and paraclinical, before and after the treatment, the data obtained being statistically compared.

### 2.2. Study Groups and Inclusion Criteria

The study included 72 consecutive cases of children aged between 6 and 14 years, who were selected and investigated between September 2021 and April 2022, after being filtered through the inclusion criteria. The study was performed within the University of Medicine and Pharmacy Craiova, the participants being selected from patients hospitalized in the Pediatric Department of the Emergency Clinical County Hospital Craiova, Romania. The inclusion criterion was represented by the presence of obesity, determined by the body mass index (BMI) ≥ 95th percentile, calculated in accordance with the World Health Organization (WHO) criteria in relation to age and gender [32]. The obese patients were divided into two groups: the group with simple obesity (SO) which was made up of patients who presented a normal liver structure on ultrasound and normal alanine transaminase (ALT) values (N ≤ 22 U/L in males and N ≤ 26 U/L in females) (only one case) or elevated ALT values (<2 N), and the NAFLD group consisting of patients with specific changes of fatty liver on ultrasound and abnormally elevated ALT values (>2 N). The distribution of patients in relation to the ultrasonographic appearance and the serum ALT value was carried out in accordance with the North American Society for Pediatric Gastroenterology, Hepatology and Nutrition (NASPGHAN) guide published in 2017, recommended by the Expert Committee on NAFLD (ECON) and approved by the European Society for Pediatric Gastroenterology, Hepatology and Nutrition (ESPGHAL) [24]. Although in recent years some authors introduced the term “metabolic dysfunction-associated fatty liver disease (MAFLD) [23] for NAFLD, the latter term was used in this study.

### 2.3. Participants

The non-probability quota sampling method was applied to select the children who met the inclusion criterion represented by obesity at the age between 6 and 14 years. The simple obesity (SO) group included 22 cases, and the NAFLD group included 50 cases. In order to ensure the homogeneity and representativeness of the analyzed groups, exclusion criteria were applied for all patients represented by the presence of autoimmune and inflammatory diseases, endocrine and hereditary diseases, and other chronic diseases or patients who had received treatments or nutritional supplements (including pro/prebiotics) in the last 6 months prior to enrolling in the study. In addition, patients who presented a high risk of non-compliance or need for the administration of treatments of any kind (nutritional, symptomatic or surgical supplements, emotional imbalances) were excluded. None of the patients had particular or non-compliant dietary habits; all patients were Caucasian and originated from the southwest of Romania—the Oltenia region.

### 2.4. Patient Assessment

In addition to the patient history that allowed the identification of ineligible cases, the epidemiological data represented by age and gender were recorded. Although there are numerous studies that addressed NAFLD in pediatric pathology, children under 10 years of age are extremely rarely investigated, especially in the absence of obvious clinical symptoms [6]. In our study, we analyzed two age groups, namely 6–9 years and 10–14 years.

The clinical and paraclinical investigation of the children included in the study addressed primarily the criteria for establishing obesity and NAFLD, namely the establishment of BMI (height, weight, age, gender), the ultrasound appearance and the serum ALT value. On the other hand, the investigations addressed the criteria for establishing the presence of metabolic syndrome (MS), which is designated as a complex clinical entity associated with the initiation and evolution of NAFLD [1,33], these being represented by the abdominal perimeter/circumference (AP), blood pressure (BP), serum triglycerides (TG), serum high-density lipoprotein cholesterol (HDLch) and glycemia. In this context, establishing the need to investigate MS associated with NAFLD was an important reason for introducing the SO group into this study.

An anthropometric investigation was performed using standardized measurement methods [34]. For height, we used a thaliometer with a graduation of 1 mm, and for measuring weight, we used a monitor for determining weight (graduation 100 g). The measurements were performed in the morning after waking up. The body mass index (BMI) was calculated by dividing the body weight (expressed in kilograms) by the square of the height (expressed in meters). All participants had a BMI ≥ 95th percentile according to the WHO reference values for the patient’s age and gender [35]. The waist circumference was measured at the end of the expiration in the umbilical area with a thaliometer for circumferences (graduation 1 mm), after which this was transformed into percentiles according to recommendations for evaluation of overweight/obese children, the percentiles above 90% being a risk factor for metabolic syndrome in the case of obese patients [36].

Blood pressure was measured three times after a rest period of ten minutes with an electronic tensiometer, and the average value was obtained. The calculation of percentiles for blood pressure values was performed according to age, gender and height using the existing software within the Centers for Disease Control and Prevention (CDC) official website [37]. For both systolic and diastolic blood pressure values, the percentile below 90% meant normal pressure; between 90% and 95%, prehypertension; between 95% and 99%, grade I hypertension; and over 99%, grade II hypertension [38]. In this study, we considered all cases in which the percentile was at least 90% to represent high blood pressure.

Imaging investigations. The patients were examined by liver ultrasonography, which confirmed the changes in the case of NAFLD: hyperechogenicity in comparison with the right renal cortex, attenuation of ultrasound waves with depth-dependent signal loss, with blurred visualization of the hepatic vascular system and dimming of the diaphragm [39]. In this study, the ultrasound investigation was used only for diagnosis and the formation of groups, without being used for their comparative study (degrees of liver damage) or depending on the treatment followed.

Serological investigations. After a 12 h fast, all study participants had blood samples collected for biochemical serum examination: ALT, TG, HDLch, glycemia and vitamin D.

The presence of metabolic syndrome (MS) was defined by the fulfillment of at least three of the following criteria: abdominal obesity, i.e., increased abdominal perimeter (AP) (≥90th percentile of waist circumference in patients of the same age and gender); high TG (≥100 mg/dL for age range 0–9 years and ≥130 mg/dL for 10–19 years); low HDLch (<40 mg/dL); high blood pressure (systolic blood pressure (SBP) ≥ 90th percentile or diastolic blood pressure (DBP) ≥ 90th percentile in children of the same age, height and gender); and fasting hyperglycemia (≥100 mg/dL) [36,40,41,42,43].

Vitamin D and intervention. Serum values of vitamin D (25(OH)D) below 30 ng/mL were considered deficient and required supplementation [44]; in this study, the term “vitamin D deficiency” refers to serum values corresponding to insufficiency (<30 ng/mL), or lower (<20 ng/mL). Patients with normal serum levels of vitamin D were trained, and the treatment consisted of imposing a specific uniform regimen, with lifestyle modification by improving nutrition and increasing physical activity: avoiding sugar-sweetened beverages, eating a well-balanced healthy diet, moderate- to high-intensity daily exercise, and less than two hours/day of screen time [24,45]. For patients with vitamin D deficiency, in addition to the regimen, the diet was supplemented with 3000 IU/day of vitamin D3 for three months in accordance with the recommendations of the guide on the evaluation and therapy of vitamin D deficiency in pregnant women, newborns and children elaborated in 2019 by the Romanian Ministry of Health [46], with this aspect being considered a mixed treatment (regimen and vitamin D). Romania is located between 44 and 48° of the Northern Hemisphere; it has a transitional temperate continental climate, specific for central Europe, with four distinct seasons, with temperature values predominantly below 10°C between October and April, and with an average length of the day during this period of 10.8 h, without major variations regarding sunny days [47]. In order to ensure the homogeneity of the group in terms of vitamin D levels and sun exposure, the administration of vitamin D was carried out between October 2021 and January 2022, as the patients were included in the study. During the study period, all patients received appropriate treatment, represented by regimen or mixed treatment (regimen and vitamin D), as they were enrolled in the study, without waiting for the formation of target groups. The legal representatives were informed about the fact that during the entire study period, the children should not be given any other treatments, with the obligation to inform the researchers about any change in the child’s health status. After three months from the initiation of the treatment, the patients were evaluated under the same conditions as at the beginning of the study, and the results were recorded to be compared. Three children from the age group of 6–9 years who in this period reached the threshold of 10 years were kept in the initial age category. Periodically, the legal representatives of the patients were called by phone to be asked about the health status and compliance of the children. For the patients included in the study, we did not find adverse effects of vitamin D administration or other medical situations that would require a review of the regimen or interruption of vitamin administration.

### 2.5. Statistical Analysis

In this study, we compared the epidemiological, clinical and paraclinical data associated with SO and NAFLD, associated or not with MS, and before and after the administration of treatment based on a specific regimen or mixed therapy (regimen and vitamin D).

The results were expressed descriptively as a number of cases and prevalence (%) for the categories analyzed. Epidemiological (age, gender), clinical (BMI, AP) and paraclinical (TG, HDLch, glycemia, SBP/DBP, ALT, vitamin D) data were tabulated into an electronic database, using Microsoft Office Excel 2010. The comparison tests within SPSS10 (Statistical Package for Social Sciences) software were used to analyze the variance of case distribution and of the measured numerical values, being represented by chi-squared (χ2), one-way ANOVA, Student’s t-distribution and Pearson tests. The results were considered significant for values of *p* < 0.05 and at the limit of statistical significance for values of *p* < 0.08. All reported numerical values are presented as average ± standard deviation.

Analysis of variance of case distribution and measured values were analyzed in tandem. Measured values were represented by serum/index values recorded for MS parameters (AP, TG, HDLch, glycemia, SBP/DBP), parameters associated with inclusion criteria (BMI, ALT) and vitamin D. The parameters used for study inclusion (BMI, ALT) were excluded for case distribution analysis. In the case of blood pressure, for the distribution of cases, we used the SBP/DBP parameter as a component of the MS; for the analysis of the measured values, we used SBP and DBP terms separately.

In order to statistically evaluate the variations in the parameters depending on the follow-up therapy, we combined the variables related to the type of lesion (SO and NAFLD) with those related to the treatment (regimen vs. mixed treatment), and we obtained four groups of patients for the comparison of the average final measured values of the parameters investigated, represented by NAFLDrd (regimen and vitamin D), SOrd (regimen and vitamin D), NAFLDr (regimen) and SOr (regimen). We considered superior the treatment in which the final average measured value of the parameters had the most improved values. The same groups of patients were compared in relation to the average values of the difference obtained by subtracting the measured initial and final values of the investigated parameters, an aspect that allowed us to evaluate the level of difference and the effectiveness of the two types of treatment. The average difference values (average level of difference) for a parameter analyzed in the SO and NAFLD groups resulted from the average of the differences for each case, differences that were obtained by subtracting the measured initial–final or final–initial values in the sense that they have improved, taking into account both positive and negative values. The more effective treatment, with faster effects, was considered the one where the average level of difference was higher.

### 2.6. Ethical Principles

The study respected the guidelines of the Declaration of Helsinki, with written informed consent being obtained from legal representatives of the children, followed by approval of the Local Ethics Committee (No. 118/19.07.2021). Each patient and relative proposed as eligible was invited to discuss the stages of the study in detail. The legal representatives were given 48 h to decide whether they agree with enrolling the child in the study.

## 3. Results

### 3.1. Initial Evaluation and Identification of NAFLD-Associated Parameters

The SO group was represented by 22 cases, with equal distribution by gender, most of them aged between 10–14 years (59.1%) and an average age at diagnosis of 9.8 ± 2 years. In this group, MS had a prevalence of 9.1%, with high AP, TG and SBP/DBP in 22.7% of cases, low HDLch in 31.8% of them, and without affecting blood glucose (Table 1). In 66.6% of SO cases, there was at least one component of MS. Vitamin D deficiency was present in 40.9% of cases.

The NAFLD group included 50 cases; most of the patients were male (64%, M/F ratio of 1.7), and most of the patients were classified in the age group of 10–14 years (56%), with an average age at diagnosis of 10 ± 2.2 years. The distribution of cases indicated an MS prevalence of 66%, with a high AP in 94% of the cases, high TG in 60%, low HDLch in 72%, high glycemia in 10% and high SBP/DBP in 54%. Vitamin D deficiency was present in 84% of cases (Table 1).

The analysis of the initial distribution of cases indicated that the age of 10–14 years was more frequently associated with NAFLD and SO, and male gender was more frequently associated with NAFLD (Table 1). At the same time, NAFLD was statistically associated with the presence of MS, the aspect being significant in the case of high AP, low HDLch and high SBP/DBP and at the limit of statistical significance in the case of high TG (Table 1). The presence of NAFLD was statistically associated with vitamin D deficiency (Table 1).

For NAFLD, analysis of the measured values of the investigated parameters (Student’s t-tests) for the two groups indicated significantly increased values of AP (*p* < 0.001), TG (*p* = 0.031), DBP (*p* = 0.001), BMI (*p* = 0.022) and ALT (*p* < 0.001) and increases at the limit of statistical significance for high glycemia (*p* = 0.077) and SBP (*p* = 0.056), as well as significantly lower values for HDLch (*p* < 0.001) and vitamin D (*p* < 0.001 ) (Table 2).

### 3.2. Final Evaluation and Overall Treatment Effect

After administration of the treatment based on regimen or mixed treatment (regimen and vitamin D), we found some changes in the distribution of cases for the two analyzed groups and in the measured values of the analyzed parameters (Table 2 and Table 3).

Thus, in the case of the SO group, MS was absent, with the presence of only high AP in 4.5% of cases and high SBP/DBP in 22.7%. For the NAFLD group, MS was present in only 2% of the cases, with different patients presenting high AP in 48% of cases, high TG in 10%, low HDLch in 8%, high glycemia in 6% or high SBP/DBP in 30% (Table 3).

After treatment, vitamin D deficiency was identified in 13.6% of SO cases and 16% of NAFLD cases.

The final distribution indicated a decrease in the prevalence of cases associated with MS components and vitamin D deficiency, for both investigated groups, with the attenuation of statistically significant differences, compared to those obtained initially, before the treatment. Thus, after the treatment, except for the statistically significant association of NAFLD with high AP, the differences for the other investigated features were non-significant (*p* > 0.005, χ2 test) (Table 3). For the whole group (SO and NAFLD), the prevalence decreased by 51.9% for high AP, 90.9% for high TG, 90.6% for low HDLch, 40% for high glycemia, 37.5% for high SBP/DBP, 97.1% for MS presence and 78.4% for vitamin D deficiency. Even through both treatments (regimen and mixed treatment) were effective, the attenuation of the parameters appeared more obvious in the case of NAFLD (Table 1 and Table 3).

However, the differences in the measured values of the investigated parameters for SO and NAFLD remained mostly significant or at the limit of statistical significance (Student’s *t*-tests): BMI (*p* = 0.042), ALT (*p* < 0.001), AP (*p* < 0.001), TG (*p* = 0.022), HDLch (*p* < 0.001), glycemia (*p* = 0.058), SBP (*p* = 0.121), DBP (*p* = 0.035) (Table 2). In the case of vitamin D, the differences in the mean serum level between the SO and NAFLD groups were non-significant (*p* = 0.142, Student’s *t*-test).

The number of cases associated with altered parameters investigated decreased simultaneously for both analyzed groups, reflected by the absence of statistical differences related to the distribution of cases, and this attenuation was at a relatively similar level, which was mirrored by keeping the statistical differences of the measured values. These results were associated with the notion of treatment in general, regardless of the type of treatment applied. It is worth noting that after the administration of the treatment (including vitamin D), there continued to be cases with vitamin D deficiency in both investigated groups. It is also interesting that AP remains statistically associated with NAFLD even after the treatment, being a more reliable parameter for NAFLD.

The effect of the general treatment (regimen or mixed treatment) used in this study could also be appreciated in a less specific way for the investigated groups by comparing the average values of the analyzed parameters before and after treatment for all 72 cases (SO and NAFLD), the differences obtained being statistically significant (Table 4).

### 3.3. The Relation of Vitamin D with the Investigated Parameters

When we did not consider the two groups (NAFLD and SO) and the type of treatment applied, we observed a different dependence of the status of the investigated parameters on the status of vitamin D. For the entire analyzed group, the treatment led to the normalization of vitamin D serum values in 84.7% of cases (Table 5). Case distribution analysis indicated a significant association of initial vitamin D deficiency with the age group of 10–14 years and male gender. After treatment, the normalization rate of vitamin D levels was higher in the 10–14 years group (80%) compared to the 6–9 years group (75%) and in males (85.9%) compared to females (68.7%) (Table 5).

At the same time, we found significant associations of vitamin D deficiency with the alteration of most components of MS, namely with high AP, high TG, low HDLch and high BBP/DBP (except for high glycemia) (Table 4). After treatment, we observed the absence of these associations (except high AP), as shown by an increase in the number of cases associated with the tendency of simultaneous normalization of the parameters and vitamin D (Table 5).

**Figure 1:**
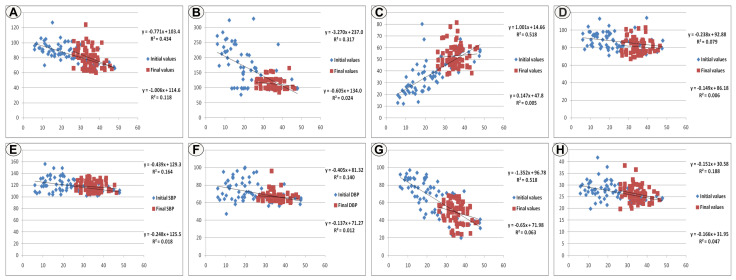
The distribution of the initial and final measured values of vitamin D in relation to AP (**A**), TG (**B**), HDLch (**C**), glycemia (**D**), SBP (**E**), DBP (**F**), ALT (**G**) and BMI (**H**).

For the whole group, the analysis of the distribution of measured initial and final values for the investigated parameters and vitamin D levels (Pearson correlation) indicated significant negative linear correlations with AP (*p* < 0.001), TG (*p* < 0.001), glycemia (*p* = 0.017), SBP (*p* < 0.001), DBP (*p* = 0.001), ALT (*p* < 0.001) and BMI (*p* < 0.001) and significant positive linear correlations with HDLch (*p* < 0.001) for the initial status (Figure 1A–H). For the final status, we recorded significant negative linear correlations with AP (*p* = 0.003) and ALT (*p* = 0.033); a correlation at the limit of significance with BMI (*p* = 0.066); non-significant negative correlations with TG (*p*= 0.191), glycemia (*p* = 0.517), SBP (*p* = 0.260) and DBP (*p* = 0.354); and a non-significant positive correlation with HDLch (*p* = 0.556) (Figure 1A–H). The intersection of the initial and final trends had the significance of the presence of an effect of vitamin D status on the measured values of the analyzed parameters. It should be noted that if the initial and final measured values of vitamin D were compared combined with those of the investigated parameters, all the correlations were statistically significant (*p* < 0.001, Pearson testing), keeping the trends of the separate evaluations, which we consider to have brought additional information.

These results indicate that the increase in the measured values of vitamin D is associated with a decrease/increase (in the case of HDLch) in the measured values of the parameters, which was sufficient (AP, ALT), borderline (BMI) or insufficient (TG, HDLch, glycemia, SBP/DBP) for statistical correlation.

The statistical insufficiency is explained by the results: As it can be seen in Table 3 and Table 5, after the treatment, a number of cases with vitamin D deficiency (15.3%) remained, along with a variable number of cases with high AP (34.7%), high TG (6.9%), low HDLch (5.5%), high glycemia (4.1%) and high SBP/DBP (27.8%). In addition, the decrease in the level of vitamin D in the cases with treatment based only on the regimen also contributed to decreasing the statistical power; thus, when comparing the differences in the initial and final measured values of vitamin D for patients who only benefited from a specific regimen (without vitamin D deficiency), we found an average decrease in the measured value of vitamin D of 1.9 ± 3.5 ng/mL in the case of NAFLD, while we found an average decrease of 0.1 ± 1.5 ng/mL for SO.

Nevertheless, the measured values indicate a direct association of the initial status of vitamin D with the status of all investigated parameters, as well as a direct association of the final status of vitamin D (after treatment) with the status of BMI, ALT and AP. By extension, taking into account the distributions and the measured values obtained (Table 1, Table 2, Table 3, Table 4 and Table 5), it can be said that vitamin D deficiency is associated with NAFLD and MS and the normalization of vitamin D is closely related to some parameters, such as BMI and ALT (also used as inclusion criteria), but especially AP, which seems to be a very strong reflection of NAFLD, as we suggested above. In the context in which we did not consider the type of treatment and groups, it can be said that the improvements in BMI, ALT and AP were specific to another specific common feature common of the group, which responded to the treatment and which can be represented by obesity.

When we took into account the two groups (NAFLD and SO) and the type of treatment applied (regimen and mixed treatment), for the measured values, we obtained variable statistical correlations between the vitamin D status (initial and final combined) and the status of the parameters (initial and final combined) for the four groups represented by NAFLDrd, NAFLDr, SOrd and SOr (Table 6). For the analysis, we took into account that in the case of NAFLD and SO, with the treatment based only on the regimen, the measured average values of vitamin D decreased by 18.3 ± 6.2 ng/mL and those of the mixed treatment increased by 10.3 ± 27 ng/mL.

For the NAFLD and SO groups, under the administration of the mixed treatment (regimen and vitamin D), we observed statistically significant linear correlations or correlations at the limit of significance of vitamin D with the analyzed parameters, with trends towards their improvement; although the improvement trend existed, we did not find a correlation for SBP and BMI in the case of NAFLD, and this result could also have the meaning of a greater dependence on the regimen. In addition to the regimen, the observed increase in the serum level of vitamin D improved the analyzed parameters.

On the contrary, for the same groups, under the administration of only the regimen, we found that most correlations of vitamin D with the analyzed parameters were statistically non-significant, with the exception of ALT for SO. The decrease in the serum level of vitamin D observed, although it does not have a statistical influence on the investigated parameters, through the recorded trends, has the potential to worsen the values of HDLch, SBP and BMI in NAFLD and PA, TG, SBP and BMI in SO (Table 6).

### 3.4. Evaluation of Effects Depending on the Type of Treatment

**Figure 2:**
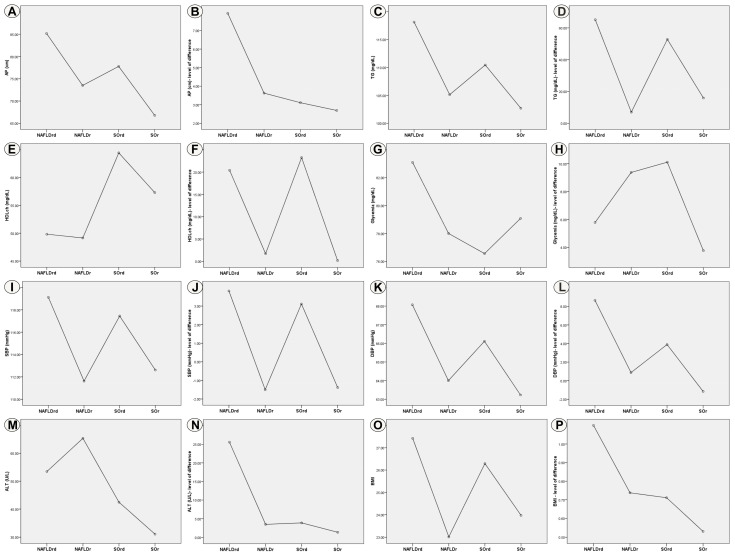
The level of the final measured mean values and the level of difference mean values for AP (**A**,**B**), TG (**C**,**D**), HDLch (**E**,**F**), glycemia (**G**,**H**), SBP (**I**,**J**), DBP (**K**,**L**), ALT (**M**,**N**) and BMI (**O**,**P**).

When we took into account the two groups (NAFLD and SO) and the type of treatment applied (regimen and mixed treatment), we found that the final measured average values and the average difference levels, for the investigated parameters, varied depending on the treatment (Table 7 and Table 8, Figure 2).

Thus, in the case of some MS components (AP, TG) and BMI, we found superior effects of the regimen-based treatment for both NAFLD and SO groups, an aspect that was based on the significantly lower statistical differences of the final measured mean values of the investigated parameters (Table 7, Figure 2). On the other hand, for these parameters, for both NAFLD and SO groups, the level of difference mean values (before and after treatment) were significantly higher in the cases where the mixed therapy was administered (Table 7 and Table 8, Figure 2), mainly due to vitamin D normalization tendency. In these cases, it was expected that the lowest final measured values would be recorded in the case of regimen-based therapy, where the alteration of parameters was milder (linear trends of Pearson tests). In the same sense, the alteration levels of the investigated parameters were higher in the case of vitamin D deficiency, and its correction had the most important effects. The results indicate once more the relation of vitamin D with the parameters investigated.

The negative values of the difference level for SBP and vitamin D in NAFLD and SO and for DBP in SO had the significance of worsening the status of the parameters in the case of the regimen, the mixed treatment being superior (Table 7 and Table 8, Figure 2).

In the case of HDLch, we found significantly superior effects and differences of the mixed treatment for NAFLD and SO (Table 7 and Table 8, Figure 2). For glycemia, we observed in the case of NAFLD non-significant superior effects in the case of regimen-based treatment, while for SO, the mixed treatment showed these improvements (Table 7, Figure 2). For ALT, the effects of the mixed treatment were superior in NAFLD, while the regimen had better effects in SO (Table 7, Figure 2). For vitamin D, the mixed treatment was superior for both groups (Table 7, Figure 2). For ALT and vitamin D, the differences were significantly higher or at the limit of significance in the case of mixed therapy for both NAFLD and SO (Table 7).

The regimen-based treatment resulted in the most improved final measured values for some of the parameters in both groups (AP, TG, BMI) or NAFLD (glycemia, DBP), but this was valid for patients without vitamin D deficiency, especially for SO cases, with the investigated parameters being less altered; the final effect was of improvement, although of relatively low amplitude. In these cases, the level of vitamin D and some parameters (SBP/DBP) worsened (Table 7 and Table 8, Figure 2).

The mixed treatment resulted in the most improved final measured values for HDLch, SBP, blood glucose (SO), DBP (SO) and ALT (NAFLD), and this was especially valid for patients with vitamin D deficiency, mainly for NAFLD cases with intensely altered investigated parameters, with the final effect of a clear improvement, spectacular and statistically significant for all parameters (except glycemia). Based on the higher average levels of absolute difference values and clinical wellbeing, improving most of the parameters, it can be said that the mixed treatment was more effective.

We obtained particular relations of some components of MS with vitamin D status. Thus, the absence of a statistical relationship of the distribution of high-glycemia patients in the analyzed groups with the status of vitamin D and the type of treatment indicates this parameter as being a component of MS with a degree of independence from NAFLD. HDLch, SBP and DBP are dependent on the administration of vitamin D. At the same time, AP seems to be the most accurate reflection of NAFLD as a component of MS.

In summary, the vitamin D deficiency and the effects of the treatments indicated a statistical dependence on the prevalence of the MS components within the SO and NAFLD groups, suggesting a pathogenic sequence.

## 4. Discussion

There are large differences in the prevalence of NAFLD in children, as reported by recent studies compared to those of 5–10 years ago [7,9], and this primarily indicates a permanent increase over the years. These discrepancies may be due to the heterogeneity of the criteria used, as well as the mixed groups of children and adolescents [14]. In addition, NAFLD is more frequent in males after the age of 10 [2,11,48,49], in a ratio of almost 3:1 with females [2], possibly due to the accumulation of especially visceral fat in males, compared to females who could also have hormonal protection as they grow older [2,18].

In our study, we found an association of NAFLD and SO with the age group of 10–14 years, as well as the predominance of males in the case of NAFLD. It is still important that the 6–9 years age group represented over 40% of the analyzed cases, which supports the need for persistent investigation of these children in order to identify obesity, NAFLD and MS. However, more and more obese children with NAFLD meet MS criteria [2,50].

Regarding the relationship between obesity, NAFLD and MS, there is a paucity of longitudinal studies in children, the aspect being viewed differently in the literature [2,10]. Thus, some authors have indicated that NAFLD is associated with MS [4,50], induces MS more than 3 times more frequently [13], is more common in those with MS [51], or is potentiated by the alteration of carbohydrate metabolism [52]; meanwhile, obesity increases the risk for MS 3 times [10], being an entity distinct from MS and not considered the initiator [12]. Our study indicated clear differences in the initial distribution of cases and the measured values of the clinical and paraclinical parameters between the two analyzed groups, with NAFLD being associated with their alteration.

Although NAFLD is considered a hepatic manifestation of MS [22] and appears 5 times more frequently in MS [51], some authors have suggested that it is actually a precursor of MS [53]. In our study, the presence of MS components in SO, which did not meet the NAFLD criteria, does not necessarily support this hypothesis. Other authors indicate MS as a comorbidity of NAFLD [11].

In this study, we found a prevalence of MS of 66% in the NAFLD group and 9.1% in SO. Besides this aspect, in the case of SO, there were cases (66.6%) that had associated components of MS, but which were not sufficient to define the syndrome. The existence of MS in the two investigated groups indicates the existence of the pathogenic sequence SO–NAFLD, which has associated components of MS and which supports their evaluation also in the case of obese children, without obvious clinical manifestations. The results support SO as a precursor entity of NAFLD.

Other studies indicate a prevalence of MS of 19.6% in the case of SO and of 29–66% in the NAFLD group [2,54]. The definition of MS in children has faced major problems over time, and only late in 2009 was it proposed to be taken into account in 6–9-year-old obese children with risk factors, which can present a series of limits if they are to be analyzed [12]. Currently, the need for a clear definition of MS components adapted to the place of origin, gender, age, ethnicity, weight and height is suggested [14].

In our study, both as a distribution of cases and as measured values, MS components are associated with the NAFLD group, compared to SO, namely high AP in 94% of cases, high TG in 60%, low HDLch in 72%, high glycemia in 10% and high SBP/DBP in 54%. However, in the distribution of cases, some components of MS appear to have variable associations with NAFLD, so while AP appears to have the greatest dependence, glycemia appears to be statistically independent. Comparatively, in a large study conducted on 254 children, Patton HM et al. identified hyperglycemia in 12.2% of cases, hypertension in 45%, low HDLch in 26% and high TG in 26% [33]. In another study with 386 children with NAFLD, hypertension was present at diagnosis in 36% of the cases [55], and for a group of 120 children with NAFLD, high TG levels were identified in 63% of the cases and low HDLch was identified in 45% of the cases [56]. Finally, in a study that included children and adolescents and in which the diagnosis of NAFLD was made by liver biopsy, high TG was found in 63% of the cases, low HDLch in 45%, hypertension in 40% and altered tolerance to glucose in 10% [16,54].

Alteration of glucose, insulin, TG, HDLch and LDL values and cardiovascular problems are also indicated in other original studies or meta-analyses on MS from NAFLD [2,4,10,11,18,50,51].

Regarding the relationship between obesity, NAFLD and MS, the literature describes factors that interfere with the pathogenic mechanisms, and among them, the role of vitamin D is also detailed.

Vitamin D is synthesized in the skin and is considered an antioxidant fat-soluble steroid hormone [8], involved in the autocrine and paracrine modulation of immunity–inflammation [4], improving lipid and carbohydrate metabolism [13,28] and improving liver markers and histopathology in NAFLD [37], as well as weight and BMI [28].

The potential mechanisms through which vitamin D exerts its pleiotropic effects are multiple and described in original studies, reviews or meta-analyses. Thus, Barchetta I et al. describe in detail the pathogenic ways of action of vitamin D and the effects of deficiency, which act in the sense of disrupting liver and intestinal homeostasis, decreasing insulin sensitivity and lipotoxicity, through extremely complex and interconnected mechanisms in which vitamin D receptors, adipocyte plasticity, microbiota and intestinal permeability play key roles [23].

Some studies indicate a deficit within very wide limits in obese adolescents, namely 29–90% [57]. It is considered that children with NAFLD have a 1.2 times higher risk of having vitamin D deficiency [58], which is present in around 50–78.9% of these cases [21,49,59]. In this study, we found the presence of vitamin D deficiency in 40.9% of the SO cases and 84% of the NAFLD cases. It can be said that vitamin deficiency is associated with obesity and NAFLD.

There are numerous studies that have indicated that vitamin D deficiency is associated with obesity, the progression of NAFLD and MS [4,10,21,58,59,60], or with some of the components of MS, such as AP, TG, BP and HDL, in an independent manner [8,49,61]. In our study, the distribution of cases indicated an association between vitamin D deficiency and all MS components, with the exception of glycemia, and in the case of measured values with all components.

On the contrary, other studies have not identified relationships between vitamin D insufficiency/deficiency and the histopathological aspects of NAFLD, nor with most components of MS, obesity or BMI [21]. Some authors consider that vitamin D has an anti-inflammatory rather than an anti-obesity effect [62], and others consider that the deficit is an independent factor for NAFLD [9]. In addition, in the first meta-analysis that investigated the link between vitamin D deficiency and NAFLD in children, the authors found an association that was not preserved when analyzing subgroups based on age, region and BMI [61], while other authors failed to demonstrate a direct relation of vitamin D with NAFLD [9]. These features once again underline the heterogeneity of the results.

Active treatment of obesity, NAFLD and MS has become necessary in this context [4]. Lifestyle, translated by weight loss and physical activity, is the only treatment that has proven effectiveness on NAFLD and improves liver histology [4,6,8], even if there are no standardized recommendations in this direction [19].

There are numerous pharmacological treatments for NAFLD, which are not validated or are in trials, and which are related to the effect of polyunsaturated fatty acids, probiotics or antioxidants, such as vitamins A and E [4,11].

In the same context, there are studies that tested the effects of vitamin D supplementation in adults or children, the results being controversial, in the context of the heterogeneity of the groups and the treatment [13,23]. While some authors recorded beneficial effects with a decrease in BP, weight and BMI; an increase in glucose tolerance; a decrease in insulin resistance; and an improvement in LDL, HDL and TG [28,63] and recommended additional vitamin D administration [8,49] even with a preventive purpose [9], others found no improvements in BMI [63], liver markers [64] or liver histology [60].

However, the relation of vitamin D with the pathogenesis of NAFLD may be slightly different in the case of children, in whom the hydroxylation capacity of the liver is generally preserved [29].

In our study, the administration of regimen-based or mixed treatment led to a decrease in the number of cases associated with components of MS simultaneously in both groups, especially in the case of NAFLD. In addition, the general treatment of our group indicated that BMI, ALT and AP are the parameters by which the attenuation of obesity with vitamin supplementation can be evaluated. Normalization of vitamin D occurred with the administration of the mixed treatment, while patients who received only the regimen had a decrease in serum levels of the vitamin for both NAFLD and SO, which suggests the need for supplementation, even for patients with normal values. In addition, the results obtained do not directly support the increase in the level of efficient serum vitamin D by unlocking it at the level of adipose deposits through weight loss, a mechanism suggested in some studies, in which adipose tissue would function as a reservoir of the vitamin [29,65]. Thus, in our study, although there was an improvement in BMI, which seems largely dependent on the simple regimen, the patients who initially had normal serum values of vitamin D recorded decreases. On the other hand, this aspect may be due to the disturbance of the intestinal microbiota or the bioavailability of the vitamin released from adipose tissue, under the conditions of possible chelating mechanisms associated with obesity, NAFLD and MS.

Most of the MS components investigated, as well as ALT and BMI, showed improvement trends in the case of mixed therapy, which included vitamin D; on the contrary, in the case of the therapy based only on the regimen, these trends were worsening for many parameters. In addition, the alteration of SBP/DBP was statistically evident in the case of patients with a simple regimen. This is important because it underlines at least a potentiating role of vitamin D supplementation for the applied regimen. The results of our study support the relationship of vitamin D deficiency with the alteration of the parameters and the normalization effect of vitamin supplementation. In a study with a design similar to ours, in which patients with NAFLD received regimen treatment or regimen and vitamin D, the authors found the superiority of the mixed treatment, at least in the normalization of ALT [66].

Some components of MS, such as glycemia, presented a particular relation in this study, relatively independent of vitamin D status or treatment. However, the improvement trend after vitamin D supplementation existed. Other authors indicate controversial effects of vitamin D on glucose levels, some indicating the absence of any relationship [67]. On the other hand, in our study, the normalization trends of HDLch and SBP/DBP are dependent on the administration of vitamin D, as resulted from the distribution of cases and the superiority and effectiveness of the treatment. For some parameters, the statistical non-significance may be due to the insufficient increase in serum vitamin D or the partial/insufficient response of the parameters to this increase, but it may also be due to a lower dependence of these parameters on the vitamin D status. At the same time, the statistical results may suggest the need to investigate and correct the intestinal absorption of vitamin D in obese patients with NAFLD by stabilizing and restoring the intestinal microbiota, which can be achieved by co-supplementation with pre/probiotics [68]; in this study, patients did not use pre/probiotics, but the assessment and personalized correction of the patients’ microbiomes could improve the results obtained.

### Limits and Advantages of the Study

Although there are many limitations of this study, they can be seen as starting points for future studies: (a) The criteria for inclusion in the study, although they are widely utilized, are relatively fragile; in our study, we did not encounter crossed aspects of the inclusion criteria, namely normal ultrasound appearance and ALT values >2 N or abnormal ultrasound appearance and ALT values < 2 N, which may indicate their validity. (b) The heterogeneity of data from the literature and the original methodology did not allow us to establish a target group from the beginning. In this sense, the number of patients can be considered too small for statistically non-significant trends; however, when evaluating each case in this study, we identified patients for which the actual values of the parameters remained almost unchanged or worsened, which indicates that there were cases that did not obey the trend. (c) With all the precautionary measures, there was the possibility of deviation from the diet and physical activity regimen of some patients. (d) Issues related to possible genetic predispositions, which are aspects described in the literature and may be the subject of future studies, were not taken for analysis. (e) There are no specific published percentile data for the pediatric population in our country, and thus the particular aspects of the region where the study was conducted cannot be extracted; this remains a desideratum that is emphasized in other recent studies [14,69]. (f) Vitamin D was administered uniformly without taking BMI into account, without specifying the dose according to the serum level of vitamin D, to which is added the dependence on the bioavailability of vitamin D, which can explain the existence of a number of cases unresponsive to the treatment; according to a study, 30% of obese patients who are supplemented with vitamin D do not respond [70], which would mean that the doses are insufficient, or that there are other pathogenic mechanisms interfering with the effects of vitamin D. Nevertheless, in our study, we obtained statistical trends that can be explored in future studies. (g) The patients come from a limited area of Romania; even if they cannot be extrapolated to a larger region, there are no clear reasons for the existence of major differences related to seasonal vitamin D levels between the regions of this country. (h) We did not take into account the distribution of adipose tissue (visceral and cutaneous), the visceral being the more metabolically active; this issue is somewhat controversial because in children it seems that the cutaneous is more important, because the visceral type is not so developed [15]. In the same direction, obesity was assessed based on BMI, and the results obtained indicated AP and ALT as associated with it; thus, there is the possibility that the analysis of fat mass distribution using DXA or BIA methods will bring additional information, aspects that must be taken into account for future studies. The advantages of these methods (non-invasive, easy to use, minimal staff training) exceed the disadvantages of using them in children (effects of growth, effectiveness of prediction equations for BIA and certified technicians, maintaining immobility for DXA) [20,68].

The major advantage of this study is the fact that it included not only patients with very low levels of vitamin D but also patients with normal levels, the patients being children under and over 10 years old, which allowed the analysis of differences; in addition, the study design decreased the heterogeneity in the variables related to season, race, internal or external factors that could influence the relation of vitamin D with NAFLD in obese children. At the same time, the study design allowed the patients to benefit from treatment during the investigations, an aspect that we consider important and which is not found in the studies performed so far on children.

## 5. Conclusions

In this study, NAFLD was associated with MS components, the pathogenic sequence SO–NAFLD being frequent in the age group of 6–9 years, which supports their systematic investigation.

Vitamin D deficiency has been associated with obesity, NAFLD and the presence of MS components in children, and the correction induced a tendency to improve them, indicating at least a role for positive potentiation of the regimen by the vitamin. BMI, ALT and AP are parameters associated with obesity, while AP is closely associated with NAFLD. MS components are important for therapeutic response, with HDLch being dependent on vitamin D status, while glycemia appears to be independent.

The treatment based exclusively on the regimen determines the additional alteration of the level of vitamin D and SBP/DBP and induces trends of negative evolution for the other investigated parameters, which supports the option of vitamin D supplementation in the absence of the deficiency.

Rigorous and homogeneous future studies are needed to identify mechanisms and clinical markers that interfere with vitamin D status, to improve patient stratification criteria.

## Figures and Tables

**Table 1:** The initial distribution of SO and NAFLD cases in relation to the analyzed parameters.

Parameter/No. Cases		SO (No. Cases) (%)	NAFLD (No. Cases) (%)	*p* Value (χ2 Test)
Age (years)	6–9	9 (40.9)	22 (44)	0.807
	10–14	13 (59.1)	28 (56)	
Gender	Male	11 (50)	32 (64)	0.265
	Female	11 (50)	18 (36)	
MS	High AP	5 (22.7)	47 (94)	<0.001
	High TG	5 (22.7)	30 (60%)	0.064
	Low HDLch	7 (31.8)	36 (72)	0.001
	High glycemia	0 (0)	5 (10)	0.124
	High SBP/DBP	5 (22.7)	27 (54)	0.014
	Present	2 (9.1)	33 (66)	<0.001
	Absent	20 (90.9)	17 (34)	
Vitamin D deficiency	Present	9 (40.9)	42 (84)	<0.001
	Absent	13 (59.1)	8 (16)	

SO: simple obesity; NAFLD: non-alcoholic fatty liver disease; AP: abdominal perimeter; TG: triglycerides; HDLch: high-density lipoprotein cholesterol; SBP: systolic blood pressure; DBP: diastolic blood pressure.

**Table 2:** Initial and final measured values of the investigated parameters.

Parameter	Initial Measured Values		Final Measured Values	
	SO	NAFLD	SO	NAFLD
AP (cm)	74.1 ± 7.5	90.5 ± 11.5	71.2 ± 7.1	83.3 ± 13.7
TG (mg/dL)	136.8 ± 42.6	171.9 ± 69.2	105.8 ± 9.7	116 ± 19.4
HDLch (mg/dL)	50.6 ± 11.9	32.3 ± 13.2	60.2 ± 9.1	49.7 ± 7.7
Glycemia (mg/dL)	84.4 ± 8.5	88.6 ± 9.5	78 ± 6.9	82.2 ± 9.1
SBP (mmHg)	115 ± 8.1	120.8 ± 13	114.5 ± 6.7	117.9 ± 8.8
DBP (mmHg)	65.3 ± 6.7	74.8 ± 12.6	64.4 ± 2.5	67.4 ± 6.3
BMI	25.5 ± 2	27.7 ± 4.2	24.9 ± 1.9	26.7 ± 3.8
ALT (U/L)	38.1 ± 9.1	77.4± 10.4	35.7± 7.8	55.3 ± 7.2
Vitamin D (ng/mL)	32.9 ± 9.7	18.9 ± 8.6	35.9 ± 5	34.2 ± 4.2

**Table 3:** The final distribution of SO and NAFLD cases in relation to the analyzed parameters.

Parameter/No. Cases		SO (No. Cases) (%)	NAFLD (No. Cases) (%)	*p* Value (χ2 Test)
MS	High AP	1 (4.5)	24 (48)	<0.001
	High TG	0 (0)	5 (10)	0.124
	Low HDLch	0 (0)	4 (8)	0.172
	High glycemia	0 (0)	3 (6)	0.241
	High SBP/DBP	5 (22.7)	15 (30)	0.526
	Present	0 (0)	1 (2)	0.504
	Absent	22 (100)	49 (98)	
Vitamin D deficiency	Present	3 (13.6)	8 (16)	0.797
	Absent	19 (38)	42 (84)	

Only modified parameters were included.

**Table 4:** Statistical significance of initial and final mean values for the entire group (SO and NAFLD).

Parameter	Initial Value	Final Value	*p* Value (Student’s *t*-Test)
AP (mg/dL)	85.5 ± 12.9	79.6 ± 13.2	<0.001
TG (mg/dL)	161.1 ± 64.1	112.9 ± 17.6	<0.001
HDLch (mg/dL)	37.8 ± 15.3	52.9 ± 9.4	<0.001
Glycemia (mg/dL)	87.3 ± 9.3	80.9 ± 8.7	<0.001
SBP (mmHg)	119 ± 11.9	116.9 ± 8.3	0.023
DBP (mmHg)	71.9 ± 11.9	66.5 ± 5.6	<0.001
BMI	27 ± 3.8	26.1 ± 3.4	<0.001
ALT (U/L)	65.4 ± 20.7	49.3 ± 11.7	<0.001
Vitamin D (ng/mL)	23.2 ± 11	34.8 ± 4.5	<0.001

**Table 5:** Case distribution in relationship with vitamin D status and the parameters evaluated for all the analyzed groups (NAFLD and SO).

Parameters/*p* Values (χ2 Test)		Vitamin D—Initial Status (No. Cases) (%)		Vitamin D—Final Status (No. Cases) (%)	
		Deficiency	Normal	Deficiency	Normal
Age	6–9	16 (22.2)	15 (20.8)	4 (5.5)	27 (37.5)
	10–14	35 (48.6)	6 (8.3)	7 (9.7)	34 (47.2)
	*p* values	0.002		0.626	
Gender	Male	35 (48.6)	8 (11.1)	6 (8.3)	37 (51.4)
	Female	16 (22.2)	13 (18)	5 (6.9)	24 (33.3)
	*p* values	0.016		0.704	
AP	High	46 (63.9)	6 (8.3)	7 (9.7)	18 (25)
	Normal	5 (6.9)	15 (20.8)	4 (5.5)	43 (59.7)
	*p* values	<0.001		0.029	
TG	High	36 (50)	2 (2.8)	0 (0)	5 (6.9)
	Normal	15 (20.8)	19 (26.4)	11 (15.3)	56 (77.8)
	*p* values	<0.001		0.325	
HDLch	Low	42 (58.3)	1 (1.4)	0 (0)	4 (5.5)
	Normal	9 (12.5)	20 (27.8)	11 (15.3)	57 (79.1)
	*p* values	<0.001		0.382	
Glycemia	High	3 (4.1)	2 (2.8)	1 (1.4)	2 (2.8)
	Normal	48 (66.7)	19 (26.4)	10 (13.9)	59 (81.9)
	*p* values	0.581		0.375	
SBP/DBP	High	29 (40.3)	3 (4.1)	3 (4.1)	17 (23.6)
	Normal	22 (30.5)	18 (25)	8 (11.1)	44 (61.1)
	*p* values	0.001		0.968	

**Table 6:** Correlations of vitamin D with the investigated parameters depending on the treatment. (+) linear positive correlation; (−) linear negative correlation.

Pearson Test (*p* Value)/Group	NAFLDrd	NAFLDr	SOrd	SOr
Vitamin D/AP	0.001 (−)	0.803 (+)	<0.001 (−)	0.692 (−)
Vitamin D/TG	<0.001 (−)	0.192 (+)	<0.001 (−)	0.678 (−)
Vitamin D/HDLch	<0.001 (+)	0.487 (+)	0.026 (+)	0.470 (−)
Vitamin D/Glycemia	0.001 (−)	0.222 (+)	0.025 (−)	0.812 (+)
Vitamin D/SBP	0.160 (−)	0.455 (−)	0.069 (−)	0.822 (−)
Vitamin D/DBP	0.007 (−)	0.090 (+)	0.075 (−)	0.427 (+)
Vitamin D/ALT	<0.001 (−)	0.535 (+)	0.002 (−)	0.024 (+)
Vitamin D/BMI	0.180 (−)	0.463 (−)	0.020 (−)	0.829 (−)

**Table 7:** The superiority and effectiveness of treatments related to the final measured values and to the level of difference.

Parameter	Treatment Superiority (Final Measured Mean Values)			Treatment Effectiveness (Level of Difference Between Initial and Final Measured Values)		
	NAFLD	SO	*p* Value (ANOVA Test)	NAFLD	SO	*p* Value (ANOVA Test)
AP	regimen		<0.001	mixed treatment		0.007
TG	regimen		0.017	mixed treatment		0.007
HDLch	mixed treatment		<0.001	mixed treatment		<0.001
Glycemia	regimen	mixed treatment	0.095	regimen	mixed treatment	0.421
SBP	mixed treatment		0.020	mixed treatment		0.108
DBP	regimen	mixed treatment	0.022	mixed treatment		0.008
BMI	regimen		<0.001	mixed treatment		0.025
ALT	mixed treatment	regimen	<0.001	mixed treatment		<0.001
Vitamin D	mixed treatment		0.078	mixed treatment		<0.001

**Table 8:** The mean level of difference between the measured initial and final values of the investigated parameters depending on the treatment.

Parameter	NAFLDrd	NAFLDr	SOrd	SOr
AP	7.9 ± 6.8	3.6 ± 3.4	3.1 ± 1.2	2.6 ± 3.8
TG	65.1 ± 65.2	7 ± 21.5	52.7 ± 25.5	15.9 ± 40.3
HDLch	20.4 ± 8.6	1.7 ± 2.3	23.2 ± 10.2	0.1 ± 4.4
Glycemia	5.7 ± 10.7	9.3 ± 11.4	10.1 ± 11.1	3.7 ± 6.3
SBP	3.8 ± 9.3	−1.5 ± 1.7	3.1 ± 5.8	−1.3 ± 5.1
DBP	8.6 ± 11.6	0.8 ± 5.6	3.8 ± 6.8	−1.1 ± 3.6
BMI	1.1 ± 0.7	0.7 ± 0.6	0.7 ± 0.4	0.5 ± 0.3
ALT	25.5 ± 10.7	3.5 ± 1.6	3.8 ± 2.1	1.3 ± 2.7
Vitamin D	18.3 ± 6.4	−0.1 ± 1.5	10.3 ± 2.7	−1.9 ± 3.5

## Data Availability

The data presented in this study are available on request from the corresponding author.

## Abbreviations

NAFLD	Non-alcoholic fatty liver disease
NASH	Non-alcoholic steatohepatitis
MAFLD	Metabolic dysfunction-associated fatty liver disease
SO	Simple obesity
MS	Metabolic syndrome
BMI	Body mass index
ALT	Alanine transaminase
TG	Triglycerides
HDLch	High-density lipoprotein cholesterol
AP	Abdominal perimeter/circumference
SBP	Systolic blood pressure
DBP	Diastolic blood pressure
NAFLDrd	Group of patients diagnosed with NAFLD treated with specific regimen and vitamin D supplementation
NAFLDr	Group of patients diagnosed with NAFLD treated with specific regimen
SOrd	Group of patients diagnosed with SO treated with specific regimen and vitamin D supplementation
SOr	Group of patients diagnosed with SO treated with specific regimen
